# Health-related quality of life: A neglected aspect of pulmonary tuberculosis

**DOI:** 10.4103/0970-2113.59259

**Published:** 2010

**Authors:** Ashutosh N. Aggarwal

**Affiliations:** *Department of Pulmonary Medicine, Postgraduate Institute of Medical Education and Research, Chandigarh, India. E-mail: ashutosh@indiachest.org*

Tuberculosis is one of the leading causes of mortality and morbidity around the world, infecting approximately 8 billion people, with an annual death rate of close to 1 million.[1] India shares almost a third of this global tuberculosis burden. With nearly 2 million incident cases and half a million deaths annually, tuberculosis is certainly an enormous public health problem in this country.[1,2] The Revised National Tuberculosis Control Programme (RNTCP) devotes considerable attention to diagnosis and therapy of disease using directly observed treatment short-course (DOTS), and a sizeable amount of research is focused on evaluation of strategies for the treatment and prevention of tuberculosis. In general, the programme data in India focuses on outcomes such as mortality and bacteriologic markers of response. However, in addition to clinical symptoms, a tuberculosis patient needs to deal with several physiological, financial, and psychological problems. The symptoms and clinical burden of disease often extend beyond the duration of treatment. Tuberculosis in India also carries a social stigma due to the perceived consequences of infection. Further, the treatment itself may be related with several side-effects. All these aspects of disease and its management have a huge impact on the overall well-being of the patient and the burden of these factors can equal and even exceed the physical impact of illness.[3]

According to the World Health Organization, health is defined as a state of complete physical, mental, and social well-being and not a mere absence of disease or infirmity. The impact of any disease, especially a chronic illness like tuberculosis, on an individual patient is therefore often all-encompassing, affecting not only his physical health but also his psychological, economic, and social well-being. In medical practice, the accepted method of assessing change among patients has been to focus on laboratory or clinical tests. Although these results provide important information regarding the disease, it is often impossible to separate the disease from the individual's personal and social context, especially in chronic and progressive diseases.[4] Kaplan and Bush proposed the use of the term “health-related quality of life” (HRQoL) to distinguish health effects from other factors influencing a subject's perceptions (such as environmental factors or job satisfaction) and constituting a complex, multidimensional construct.[5] One must deviate from the traditional indicators of disease severity and treatment response to capture the overall health status, with a greater emphasis on patient's, rather than clinician's, perspective of disease. An objective assessment of patient's HRQoL represents the functional effects of an illness and its consequent therapy on a patient, as perceived by the patient. HRQoL measures are, however, not a substitute for disease outcomes, but are an adjunct to them. Medical interventions may result in improved functional health status without evidence of physiologic improvement and vice-versa. Several generic and disease-specific questionnaires are now available for quantifying HRQoL in patients with a wide variety of clinical disorders. Almost all instruments have been developed and validated in Western societies and patient groups. The appropriateness of existing HRQoL measures in India is therefore uncertain.

Unfortunately, little attention has been paid to the impact of the burden of illness and its therapy on the HRQoL of patients with tuberculosis. A review of the English literature identified only 60 articles addressing one or more aspects of HRQoL in patients of tuberculosis.[6] This review could not retrieve any study that had utilized standardized generic or disease-specific HRQoL instruments in these patients. More recently, there have been reports on the use of such standardized instruments to assess HRQoL in patients of tuberculosis.[7–9]

Somatic symptoms reflect patient's physical sensations as a result of disease or its treatmen, and are the most extensively studied HRQoL domain of tuberculosis. However, in most such studies, it is not clear whether the symptoms described were spontaneously reported by patients or elicited by clinicians.[10] The range of symptoms of tuberculosis is broad and patients may report no symptoms or specific single-organ complaints, or present with life-threatening manifestations. The most commonly reported symptoms are fever and cough, which are more common in men and middle-aged individuals.[11,12] With treatment, symptomatic improvement begins in 2–3 weeks. Persistence of symptoms is generally higher among those who seek delayed treatment.[9]

Physical functioning reflects the capacity of the patient to carry out basic day-to-day activities. Tubercular arthritis is well associated with long-term disability.[13] The disease also moderately affects the non-job daily activities of nearly half of the patients with tuberculosis.[9]

Psychological health takes into account several facets of the individual's mood and emotional well-being. Most patients are worried, frustrated, or disappointed by the diagnosis, and almost a quarter do not initially accept their diagnosis.[9,14,15] The economic burden of illness as well as distress about spreading disease to others may also impair the pshycohological health.[16,17] These negative emotions generally decline during the course of successful antitubercular therapy.[9]

Role functioning encompasses a person's ability to function in designated roles at work, society, and home. Irrespective of their occupation, patients lose 4–10 weeks of work because of disease and its treatment.[17–19] Patients are also afraid of informing their employers about their diagnosis to avoid losing job or wages.[20] Having a tuberculosis patient in the family increases the workload on the primary caregivers (including wives and mothers), thereby reducing their capacity to generate income and care for other family members.[21] Women with tuberculosis participate less in household activities and, therefore, avoid seeking medical care until the disease is far advanced.[17,22] In India, it is also common for women with tuberculosis to be rejected by their husbands or be sent away until cured.[22]

Social functioning includes a patient's interaction with other people around him at home, work, and society. The marital impact of a diagnosis of tuberculosis is well known. It is difficult to arrange marriage for boys and, more commonly, girls, suffering from this disease. In many instances, knowledge of diagnosis has resulted in divorces or second marriages. Among patients admitted to isolation facilities, many feel lonely, bored, confined, or abandoned.[10,23] In other instances, unfriendly health care workers made some patients feel frustrated, threatened, unwelcome, or uncomfortable.[10,24] After discharge from the health care facility, many patients are not received back into their homes.[25] Even after successful treatment and cure, several patients continue to feel inhibited from visiting acquaintances and from revealing their diagnosis to colleagues or even their spouses.[9] Such discrimination against tuberculosis patients is a key determinant of non-adherence to antitubercular treatment.[26] Patients are known to provide false addresses at tuberculosis clinics to avoid stigmatization of the entire family.[14,15]

Financial well-being of individuals and families is also affected by tuberculosis and is often related to impairment in role functioning. Although antitubercular therapy is usually provided free as part of health programmes, the other costs of illness and treatment (such as loss of wages, travel to health care facilities, laboratory investigations, management of emergencies, drug-related adverse events, etc.) have to be borne by patients and/or family members.[26] In India, almost a third of patients reported that they could not afford sufficient food, clothing, or books for their children.[17] Many children of parents with tuberculosis are forced to discontinue schooling or start working to contribute to the finances. Patients and families also dig into their savings, borrow money, and sell household articles to fund treatment.[21] Patients may choose to return to work rather than continue therapy as a result of these expenses.[24] A sizeable proportion of patients (3–80%) suffer from financial constraints due to tuberculosis and the misery gets compounded further if the patient is also the sole or primary wage earner for the family.[18,27]

The data on formal assessment of HRQoL in patients of tuberculosis is rather sparse. Dion *et al*. evaluated the feasibility of using the Medical Outcomes Study Short Form-36 (SF-36), and the 5-item EuroQol questionnaire in patients with latent, active, or previously treated tuberculosis, and showed these instruments to be reliable.[8] Chamla evaluated the SF-36 during sequential assessment of 102 patients on antitubercular treatment in China and demonstrated improvement in scores over the course of therapy.[7] In India, Rajeshwari and coworkers used a modified SF-36 instrument on 602 patients receiving antitubercular drugs under RNTCP at Chennai and showed substantial impairment in HRQoL, especially among women.[9] All these studies have used generic HRQoL instruments developed in the West. A small study from Delhi recently used the Hindi version of the abbreviated World Health Organization Quality of Life instrument (WHOQOL-Bref) to quantify impairment in the HRQoL in newly diagnosed patients of pulmonary tuberculosis.[28] This generic HRQoL measure has recently been developed and validated in India for use in Indian people as part of a global initiative of the World Health Organization.[29] In addition, Dhingra and Rajpal have recently developed a disease-specific HRQoL instrument (DR-12 scale) from data on patients of tuberculosis treated under RNTCP at Delhi.[30] The DR-12 scale has 12 items over two domains – symptoms and sociopsychological/exercise adaptation, and has shown strong construct validity and responsiveness.

We have recently conducted a prospective longitudinal study on more than 1,000 patients newly enrolled for DOTS at Chandigarh and have used both WHOQOL-Bref and DR-12 scales to summarize the HRQoL in these patients at baseline, at end of the intensive phase of therapy, and at completion of therapy. Our findings suggest that HRQoL is markedly impaired across all domains in patients of pulmonary tuberculosis and improves rapidly and substantially with antitubercular therapy administered under the RNTCP (unpublished data). However, residual impairment in HRQoL, even after successful completion of treatment, is not infrequent. Our experience suggests that locally appropriate HRQoL instruments can be successfully administered under field conditions with good data quality and that these measures show satisfactory validity, reliability, and responsiveness in patients with tuberculosis. There is, therefore, a case to consider HRQoL assessment as an adjunct outcome measure for tuberculosis patients treated through RNTCP in India.
